# Spreading Information in a Network of Interacting Neighbours

**DOI:** 10.1371/journal.pone.0102801

**Published:** 2014-07-18

**Authors:** Konrad Halupka

**Affiliations:** Department of Behavioural Ecology, University of Wroclaw, Wroclaw, Poland; Universidad de Zarazoga, Spain

## Abstract

Dispersed individuals can coordinate the onset of life history events, like reproduction or migration, on a large (population) spatial scale. However, the mechanism of this synchronisation has not yet been identified. In many species signals produced by one individual stimulate signalling activity of immediate neighbours. I propose that such local focuses of signalling could transform into waves propagating in space. This hypothesis predicts that signalling self-organizes into bursts, because neighbours tend to enter activity and refractory periods together. Temporal characteristics of such pulses should be more similar in locations proximate to one another than in distant ones. Finally, denser populations should produce relatively more complex wave patterns, since the number of propagating waves is proportional to the number of individuals. These predictions were tested in an analysis of time series of numbers of territorial songs in chaffinches, *Fringilla coelebs*, and the results supported the hypothesis. Time series of singing activity had memory of their past states: they were autoregressive processes with spectra in which low frequency oscillations predominated. The degree of similarity in two synchronously sampled time series, measured as a Euclidean distance between their spectra, decreased with the increasing physical distance of sampling spots and the number of signalling males. It is concluded that networks of interacting neighbours may integrate populations synchronising life cycles of dispersed individuals.

## Introduction

Spreading of social information is important for synchronising reproduction in groups or breeding colonies [1], [2], [3], [4], [5], [6]. A similar synchronisation effect has been demonstrated for territorial species [2], [7], [8], but underlying mechanisms of information transfer are not known. Research examining animal communication networks is framed by McGregor's [9] definition ("a group of several animals within signalling and receiving range of each other"), stressing the role of direct interchange of information. Most studies to date have focused on groups of individuals aggregated in small areas of leks and breeding colonies [4], [10], or communication in groups of individuals consisting of a few neighbours [11], [12]. Transfer of information among dispersed individuals at the population spatial scale has, however, remained beyond the scope of research.

In various organisms signals produced by one individual stimulate signalling activity in conspecifics [13], [14], [15], [16], [17]. It can be hypothesised that even if each animal only exchanges information with its immediate neighbours, local focuses of signalling activity can transform into waves, which by the domino effect propagate in space [18], [19]. This may produce a complex pattern of overlapping waves of different lengths, amplitudes and phases.

Studying details of such a communication system might be difficult without a theoretical model, but some preliminary predictions can be made. First, if singing is locally synchronised, because immediate neighbours tend to enter activity and refractory periods together, signalling intensity should have a memory of its past states. Second, if local pulses of signalling can propagate further as waves, periodicity in the number of signalling individuals should have similar characteristics in distant locations. With increasing distance, however, such similarities should gradually diminish, cancelled out by waves of signalling generated in other places. Third, the number of local foci of activity should be an increasing function of the number of individuals. Thus dense populations might produce more complex patterns of propagating waves (in terms of the number of overlapping oscillations) than sparse ones. This work reports the results of tests of these predictions using time series of numbers of songs by territorial male chaffinches, *Fringilla coelebs*, a common Old World songbird [20].

## Methods

Males chaffinch advertise territories with a song in the 2–6.5 kHz frequency band (with the peak energy between 3.1 and 4.2 kHz), that is a countable, 2–2.5 s long phrase. In natural conditions a male can produce up to 10 songs per minute [21]. Naguib and co-workers [22] simulated the process of attenuation of the chaffinch song through reverberation and absorption of sound by tree trunks, and suggested that birds could hear neighbours singing at 120 m. However, the effect of wind in the canopy was not included in the model. Some noise from rustling of leaves and branches could significantly obscure the detection of signals [23], thus reducing the active space of the song.

Field work was conducted in 2012 and 2013 in SW Poland, in mature deciduous and mixed managed forests, between 19 April and 30 May. Sampling sites were localised within the quadrilateral with the following coordinates of vertices: 51.526 N & 17.264 E, 51.248 N & 16.413 E, 50.226 N & 17.011 E, 50.173 N & 16.726 E. Altitudes of sampling sites varied from 70 to 1100 m above sea level and the most distant locations were 152.9 km apart. At each sampling site, in accordance with altitude-related phenological variation, singing activity was measured at an early stage (prior to the midpoint) of the breeding season.

Observations were done 3 to 12 h after sunrise. Each session took place in a unique sampling site (more than 0.5 h after the preceding observation session and at least 500 m from the previous location) and lasted 1 hour. The observer noted the positions of singing individuals in order to estimate their number. Also, within every 1-minute interval, the observer counted all songs. The number of songs per minute could exceed 20 and males frequently changed their positions. As a result, it was impossible to count how many songs each individual produced. It appeared, however, that males did not differ much in their performance. Thus, at 16 sampling sites where songs were enumerated in the four 90° sectors around the observer, chi-square tests revealed no significant differences between the sectors (0.68<P<0.97).

Two data collecting protocols were used: ‘single sample' (one observer) and ‘paired sample' (two observers with synchronised watches). In the paired sample protocol, both observers started the session at the same time, in a homogeneous forest area (a similar species composition and average age of trees resulting from forest management practices). The distance between the paired sampling sites was measured with a hand-held GPS (WAAS-corrected) and varied from 200 to 850 m.

Altogether, within 142 h of observation, there were 30 sessions run in the single-sample and 56 sessions in the paired-sample regimes. Statistical analyses were done with the R version 2.14 [24]. Paired sample data were treated as dependent. To use as much information as possible, in analyses that required independent time series, I applied the resampling method. Thus descriptive statistics in the Table 1 were calculated as means of 10 000 subsamples including 71 time series: a randomly selected one from each of 56 matched pairs and a randomly selected half of 30 time series collected with the single-sample protocol.

**Table 1:** Descriptive statistics of 71 time series and their 95% bootstrap percentile confidence intervals (see Methods).

Variable	Mean	95% confidence interval
No. singing males	4.9	4.7–5.1
No. songs per minute	6.7	6.0–7.3
No. of songs v. time (r_s_)	−0.023	−0.057–0.013
Noise exponent of spectrum (β)*	0.934	0.888–0.981

* log(power)  = 1/log(frequency)^β^ [31]

Memory effects in time series were analysed with the ‘ar' function of R. It fits autoregressive models of order p, thereafter referred as AR_p_:
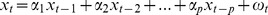
where x_t_ is the series value at the discrete t time interval, α_1,2…p_ are the model parameters and ω is the white noise. The function applies the Akaike information criterion to choose the order of the model. It returns p = 0 for random series without memory (all α values equal 0). A special case of a memoryless process is the random walk, which the function identifies as an AR_1_ model with α_1_ = 1 and other αs = 0. To verify if the particular AR_1_ series was the random walk, I used the Phillips-Perron test (‘pp.test' in R).

Cyclic components in the number of singing males were found using spectral analysis. Its premise is that a stationary time series can be reproduced by adding together a series of sine and cosine waves of particular frequency and amplitude. Sets of such component waves, called power spectra, were computed with the R function ‘spectrum', which detrends the series, applies a split cosine bell taper to 10% of the data at the beginning and the end, and calculates the fast Fourier transform. The resulting raw periodogram consisting of 30 frequency bins, was smoothed by applying a Daniell kernel with a frequency bandwidth of 0.088 per minute. The spectrum was log-transformed and scaled so that its area was one-half the variance of the time series [25]. A spectrum could be visualised as a point in multidimensional space, where each dimension is one frequency bin, and the point's coordinates are specified by amplitudes of waves of a particular frequency. Thus a degree of similarity between spectra of matched time series might be expressed as a Euclidean distance [26]. Its distribution was normalized using the Box-Cox transformation (λ = 0.38).

### Ethics Statement

The chaffinch breeds wherever trees grow, including human settlements (parks, gardens, tree alleys etc.), and thus is tolerant of the presence of humans [20]. The field part of the research depended on tallying up the number of songs uttered by males. The human observers did not interact with the birds, which sang in the canopy and thus were likely unaware of being observed. All observations were carried on in managed forests and not in nature reserves. In conclusion, according to the law of the Republic of Poland (the Animal Protection Act from 21 Aug. 1997, and the Nature Protection Act, from 16 April 2004, with subsequent changes), no special permissions were required to conduct the project described here.

## Results

**Figure 1:**
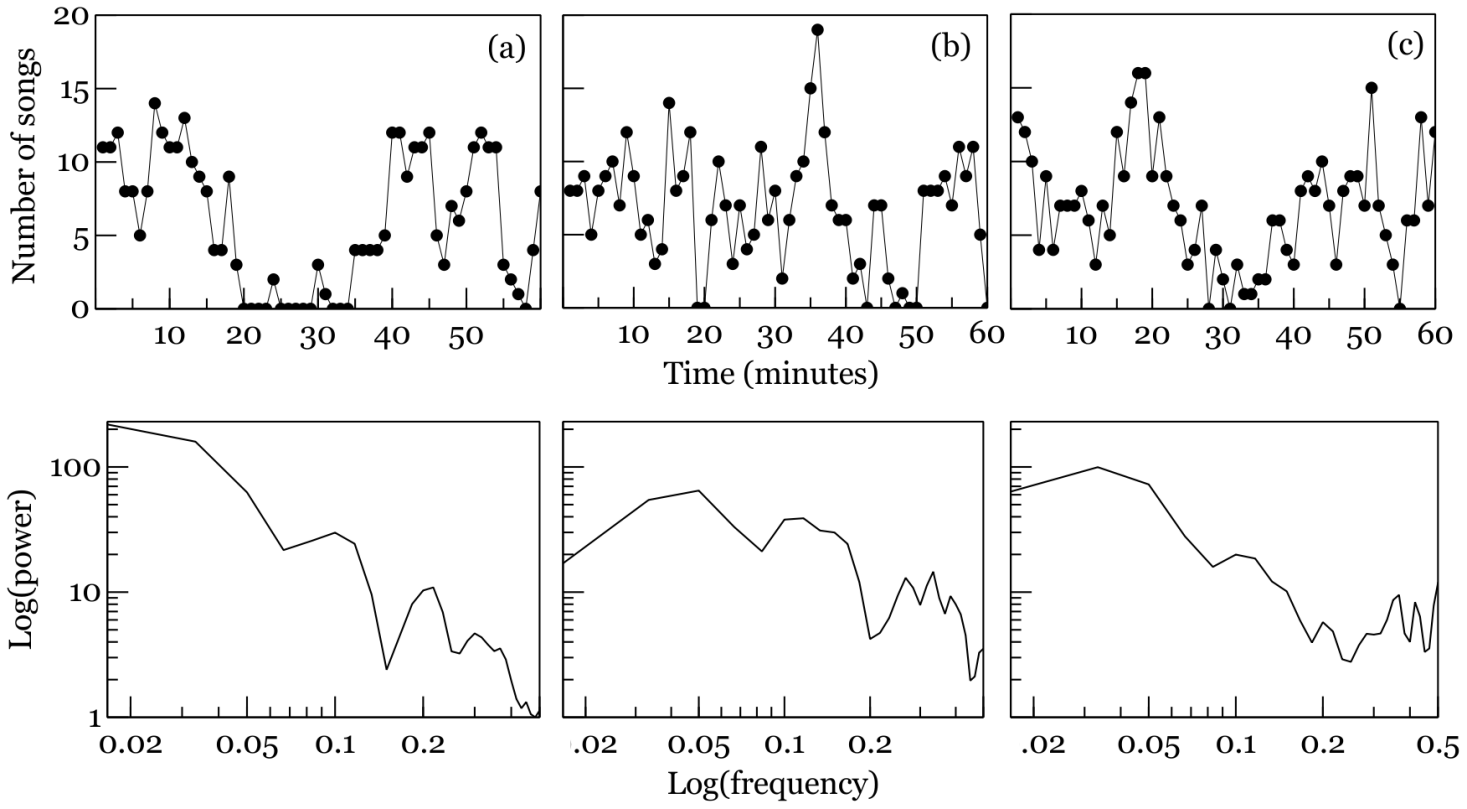
Representative time series of numbers of songs (upper panel) and their power spectra: (a) random walk, (b) mean-reversive AR_1_, (c) AR_2_.

Representative time series of numbers of songs are presented in Figure 1 and descriptive statistics in Table 1. Resampling analysis revealed that among 71 independent series there were 0.7% in which the numbers of songs in subsequent time intervals varied randomly (as in white noise), 13.4% exhibited random walk behaviour, 53.3% were mean-reversive AR_1_ series, 14.1% AR_2_, 10.6% AR_3_, and 7.9% were AR_4–8_ series. Altogether, 14.1% of the series were memoryless (white noise or random walk) and the remaining 85.9% exhibited memory. This proportion reversed when the data within each series were shuffled and the analysis was run again: 72.9% series were recognized as white noise, random walk series did not occur, and 27.1% were AR_1–9_ processes (χ_1_^2^ = 49.9702, p<0.0001).

**Figure 2:**
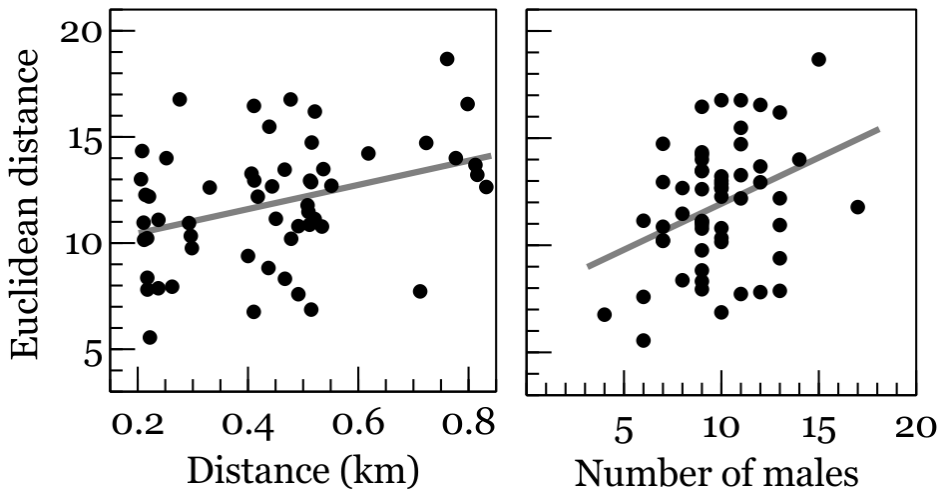
Euclidean distance between spectra of synchronously sampled time series v. physical distance between sampling points and population density. Least-square linear regression lines were fitted to illustrate trends (see Table 2 for the multiple linear regression model).

The regression model (Table 2, Figure 2) demonstrated a statistically significant tendency for a linear increase in Euclidean distance between spectra of synchronously sampled time series (N = 56 pairs) with increasing physical distance between sampling points and population density. Alternative models with quadratic or higher-order polynomial terms of predictor variables produced insignificant beta coefficients (p>0.6).

**Table 2:** Multiple regression analysis of the Euclidean distance between spectra of synchronously sampled time series v. physical distance between sampling locations (in kilometres) and the sum of individuals that were active at both places (a proxy of population density).

Predictor	Coefficient	SE	t	p
Intercept	6.4193	1.6026	4.006	<0.001
Distance in space	4.6282	2.0136	2.298	0.026
Number of males	0.3435	0.1539	2.233	0.030

The model explained 17.3% of variance in the Euclidean distance of spectra (F_2,53_ = 6.742, p = 0.002).

## Discussion

The hypothesis explaining the mechanism of information transfer when signallers-receivers are dispersed, proposes that on the local level signalling tends to self-organize into pulses, which may spread as waves encompassing the entire population. Such phenomena have been reported previously in group-living animals. Beauchamp [18] recorded and analysed characteristics of "collective waves of sleep" in groups of resting gulls (*Larus sp*.), and a synchronisation of sexual advertising by impromptu coalitions consisting of several neighbouring males, was discovered in colonies of fiddler crabs (*Uca sp.*), where the signalling wave preceded the passing female ("males behind her drop out of the group and males in front of her join in"; [27]). More recently, Kastberger and co-workers [28], described collective defence behaviour in giant honeybees (*Apis dorsata*): generator bees in the centre of the nest raise their abdomen and their nest mates around follow them in sequential order, which creates a kind of Mexican wave spreading within the hive. A similar, Mexican-wave behavioural pattern, was found among signalling black-tailed prairie dogs (*Cynomys ludovicianus*) in assessing collective vigilance of their colony members [19]. It might be argued, that the difference between gregarious and territorial species in the potential for the "globalization" of behaviour, might be purely quantitative: in the former each individual can observe many conspecifics, whereas in the latter, information about their behaviour is limited to a few immediate neighbours, as a result of dispersal. This obstacle might be overcome, however, if the signals employed spread widely, and in songbirds the frequency structure and amplitude of territorial songs are adapted to indicate an individual's presence throughout territories of its neighbours [29].

Time series of numbers of songs had autoregressive properties and low frequency oscillations prevailed (reddened spectra), manifesting memory of the past states of the system [30], [31]. The further analysis also revealed that an average β exponent in log-log spectra was close to one (Table 1), which is characteristic for self-organizing critical phenomena [32]. In such systems the action at some place depends on events happening long before at distant places. The "critical point" in the system analysed here might be the network's transition from chaos, when all individuals signal independently, into the ‘conductive' state, when neighbours coordinate signalling and information spreads. Another theoretically possible extreme state might be an absolute order with only a single, predictable wave. Something close to this scenario would occur if the wave was generated at a single spot and all (equal) males sang only in response to social stimulation. In real settings, however, males also spontaneously start singing in dyadic territorial disputes [33] or to allocate energy to sexual advertising [34]. Newly created seeds of activity can potentially generate new waves, if other individuals join in. In sum, the network of interacting neighbours might be continuously driven to the critical state by the two parallel processes: a tendency of males to respond to social stimulation and a random delivery of new signalling pulses due to spontaneous activity of individuals.

The Euclidean distance between spectra of synchronously sampled time series increased with increasing population density. This might be explained as an effect of the combination of a higher number of generated waves and their more frequent overlap, which produces new waves resulting from interference. The Euclidean distance of spectra also linearly increased with the physical distance between sampling locations. The distances between the observation sites ranged from 200 m, at which some males in both locations could possibly hear each other (considering that the active space of the chaffinch song is about 100 m; see Methods), to 850 m, when the two groups of observed males were separated by several territories occupied by other individuals. I had expected a curvilinear relationship, with the slope of regression line flattened out at longer distances between sampling points. Nevertheless a linear function best characterised the relationship between distance and spectral similarity, suggesting that ordering of similarities in the rhythm of signalling along the gradient of physical distance was fairly strong.

The network of interacting neighbours may allow dispersed individuals to avoid fitness costs of asynchrony of life cycles [35], [36]. Networking also could be regarded as a way of ‘voting' that involves a large number of participants, thus potentially increasing the accuracy of their decisions [37], [38]. This might be especially important in the tropics where, given the absence of distinct environmental cues, the start of the breeding season is "negotiated" [7], [39]. At high latitudes, where photoperiod is a reliable indicator of phenological stage, diffusion of information between individuals may fine-tune the onset of a new life-history stage such as breeding or migration [40], [41].

The idea of population integrity achieved through transfer of signals might be considered as a process of maintaining some kind of a common good by group selection. Socially stimulated signals are selected for, however, since they have other important functions directly related to individual fitness [13], [15], [16], [17]. In songbirds, territorial signalling is a form of ritualised aggression: the male is motivated to sing when its neighbours do, because otherwise he risks an escalation of conflict, when other males try to trespass [42]. Thus the population-scale network of signallers could be treated as an epiphenomenon emerging from local territorial disputes. In other animals using acoustic signals, local interactions may depend, at least in part, on other selection pressures, for example competition between vocalising males due to the so called ‘precedence effect' [17]. Generally, whenever local dynamics produces signalling clustered in time, the emerging global network of interacting neighbours might be similar.

In summary, the results presented here suggest that temporal patterns of signalling activity have a tendency to spread, even if individuals are dispersed. To explain details of the process of formation and propagation of signalling pulses, including their ranges and speeds, future studies should adopt a more sophisticated research design, ideally a network of sound recorders working synchronously and distributed over a large area. Correlative studies based on spectral analysis of time series might be complemented by an experimental approach. For example, a burst of signalling activity could be elicited by the playback. Then the movement of the wave of signalling could be followed by the grid of recorders.
